# Editorial: Redox Physiology in Fish

**DOI:** 10.3389/fphys.2026.1829914

**Published:** 2026-03-31

**Authors:** Slađan Pavlović, Gisele Cristina Favero, Mustafa Canli

**Affiliations:** 1 Department of Physiology, Institute for Biological Research “Siniša Stanković” - National Institute of the Republic of Serbia, University of Belgrade, Belgrade, Serbia; 2 Aquaculture Laboratory, Department of Animal Science, School of Veterinary Medicine, Federal University of Minas Gerais, Belo, Horizonte, Brazil; 3 Faculty of Arts and Sciences, Department of Biology, Çukurova University, Adana, Türkiye

**Keywords:** antioxidant defense, cell signaling, fish, homeostasis, oxidative stress, pollution, prooxidant/antioxidant status, xenobiotics

Aquatic organisms constantly interact with a dynamic and often challenging environment. Among the many physiological processes involved in their adaptation to environmental changes, the regulation of cellular redox balance plays a central role. In fish, the equilibrium between the formation of reactive oxygen species (ROS) and the activity of antioxidant defense mechanisms is essential for maintaining cellular stability. When this balance shifts toward excessive oxidant production, oxidative stress may arise, leading to damage of biological macromolecules such as lipids, proteins, carbohydrates, and nucleic acids. These alterations can ultimately compromise cellular function and organismal health (Lushchak, 2011).

The increasing influence of human activities on aquatic ecosystems has intensified interest in the mechanisms by which fish respond to environmental stressors. Chemical contaminants, emerging pollutants, and fluctuations in environmental conditions may all disrupt redox homeostasis. In response, fish activate a range of physiological and biochemical defense strategies designed to limit oxidative damage and maintain metabolic balance. Investigating these responses provides important insights into the health status of aquatic organisms and offers valuable tools for environmental monitoring (Grădinariu et al., 2025).

Metallothioneins, low molecular weight proteins rich in cysteine, play key roles in regulating and mitigating oxidative stress in fish. They bind metal ions such as zinc, copper, and cadmium through thiol (–SH) groups, contributing to metal homeostasis and detoxification. Under oxidative stress, metallothioneins act as effective antioxidants by scavenging reactive oxygen species and preventing metal-catalyzed formation of highly reactive radicals. Cysteine residues are central to cellular redox regulation due to their high reactivity and ability to undergo reversible oxidation and reduction reactions. In fish tissues, cysteine-containing molecules such as glutathione interact closely with metallothioneins, enhancing the overall antioxidant defense system. The coordinated action of metallothioneins and cysteine protects cellular macromolecules from oxidative damage, supports redox balance, and serves as a sensitive biomarker system for assessing oxidative stress and metal exposure in aquatic organisms (Atli and Canli, 2008).

The articles collected in this Research Topic contribute to the growing body of knowledge on oxidative stress physiology in fish exposed to diverse environmental challenges. The studies address several important aspects of redox biology, including pollutant-induced oxidative stress, physiological consequences of contaminant exposure, and the potential protective effects of natural bioactive compounds. Together, these contributions highlight the importance of integrating molecular, biochemical, and physiological approaches in order to better understand the responses of fish to environmental pressures.

A number of studies examine the impact of nanomaterials, such as titanium dioxide nanoparticles (TDNPs), whose increasing industrial use raises concerns for aquatic life. Behairy et al. evaluated TDNP toxicity in backwards Nile tilapia (Oreochromis niloticus) and the mitigating role of α-sitosterol (STL). TDNP exposure led to reduced body mass, impaired hepatic and renal function, and altered hematological parameters. It also induced oxidative stress, endoplasmic reticulum stress, and increased autophagy in the liver. Dietary STL supplementation significantly reduced these adverse effects, suggesting this phytosterol acts as an antioxidant and immunomodulatory compound capable of alleviating nanoparticle-induced toxicity.

Another contribution focuses on the neurotoxic potential of silver nanoparticles (AgNPs), using zebrafish (*Danio rerio*) as an experimental model. Yan et al. investigated how exposure to AgNPs affects larval development and behavior and examined whether bovine serum albumin (BSA) could influence these effects. Their results indicated that AgNP exposure significantly reduced locomotor activity and interfered with normal development of both the cardiovascular and nervous systems. The observed abnormalities were linked to disruptions in neuronal differentiation and axonal development. Interestingly, the presence of BSA in the exposure medium markedly attenuated the neurotoxic effects of AgNPs. This protective influence may be explained by interactions between BSA and nanoparticles that modify particle size and surface properties, thereby limiting their uptake by biological tissues. These findings emphasize that interactions between nanoparticles and naturally occurring organic molecules should be considered when evaluating the ecological risks of nanomaterials.

Traditional pollutants remain another major concern for aquatic ecosystems. Among these, pesticides are frequently detected in freshwater habitats and may have significant biological effects on fish. Yıldırım et al. examined the toxicological consequences of exposure to the insecticide flupyradifurone (FPF) in juvenile common carp (*Cyprinus carpio*). Their results demonstrated that exposure to this compound produced dose-dependent physiological responses during a 14-day experimental period. Hematological measurements revealed reductions in erythrocyte numbers, hemoglobin levels, and hematocrit values, indicating impaired oxygen transport capacity. Biochemical analyses also showed increased glucose concentrations and elevated activities of liver enzymes, suggesting metabolic disturbance and possible hepatic stress. In addition, comet assay analysis revealed clear evidence of DNA damage at higher exposure concentrations. These findings demonstrate the usefulness of combining physiological and molecular biomarkers when evaluating the environmental impact of pesticide contamination.

Heavy metals continue to represent persistent pollutants in many aquatic environments. Cadmium, in particular, is well known for its toxic effects on aquatic organisms. In this Research Topic, Zhang et al. investigated the impact of cadmium exposure on juvenile genetically improved farmed tilapia (GIFT, *Oreochromis niloticus*). The study evaluated several physiological and molecular parameters, including growth performance, digestive enzyme activity, muscle composition, and gene expression related to antioxidant defense and lipid metabolism. Cadmium exposure significantly affected growth and metabolic processes, while also altering the expression of genes associated with oxidative stress responses. These findings highlight the vulnerability of juvenile fish to heavy metal exposure and underline the importance of maintaining good water quality during early developmental stages.

Overall, the contributions gathered in this Research Topic expand our understanding of how fish respond to environmental stressors that disturb redox homeostasis. By combining physiological observations with biochemical and molecular analyses, these studies provide new insights into the mechanisms underlying oxidative stress responses in aquatic organisms. Such knowledge is essential for improving environmental monitoring strategies, strengthening ecological risk assessment, and supporting sustainable management of aquatic ecosystems and aquaculture production.

